# "I'm on it 24/7 at the moment": A qualitative examination of multi-screen viewing behaviours among UK 10-11 year olds

**DOI:** 10.1186/1479-5868-8-85

**Published:** 2011-08-03

**Authors:** Russell Jago, Simon J Sebire, Trish Gorely, Itziar Hoyos Cillero, Stuart JH Biddle

**Affiliations:** 1 Centre for Exercise, Nutrition & Health Sciences, School for Policy Studies, University of Bristol, 8 Priory Rd, Bristol, BS8 1TZ, UK; 2 School of Sport, Exercise & Health Sciences, Loughborough University, UK; 3 Nursing Department, University of the Basque Country, Spain

## Abstract

**Background:**

Screen-viewing has been associated with increased body mass, increased risk of metabolic syndrome and lower psychological well-being among children and adolescents. There is a shortage of information about the nature of contemporary screen-viewing amongst children especially given the rapid advances in screen-viewing equipment technology and their widespread availability. Anecdotal evidence suggests that large numbers of children embrace the multi-functionality of current devices to engage in multiple forms of screen-viewing at the same time. In this paper we used qualitative methods to assess the nature and extent of multiple forms of screen-viewing in UK children.

**Methods:**

Focus groups were conducted with 10-11 year old children (n = 63) who were recruited from five primary schools in Bristol, UK. Topics included the types of screen-viewing in which the participants engaged; whether the participants ever engaged in more than one form of screen-viewing at any time and if so the nature of this multiple viewing; reasons for engaging in multi-screen-viewing; the room within the house where multi-screen-viewing took place and the reasons for selecting that room. All focus groups were transcribed verbatim, anonymised and thematically analysed.

**Results:**

Multi-screen viewing was a common behaviour. Although multi-screen viewing often involved watching TV, TV viewing was often the background behaviour with attention focussed towards a laptop, handheld device or smart-phone. There were three main reasons for engaging in multi-screen viewing: 1) tempering impatience that was associated with a programme loading; 2) multi-screen facilitated filtering out unwanted content such as advertisements; and 3) multi-screen viewing was perceived to be enjoyable. Multi-screen viewing occurred either in the child's bedroom or in the main living area of the home. There was considerable variability in the level and timing of viewing and this appeared to be a function of whether the participants attended after-school clubs.

**Conclusions:**

UK children regularly engage in two or more forms of screen-viewing at the same time. There are currently no means of assessing multi-screen viewing nor any interventions that specifically focus on reducing multi-screen viewing. To reduce children's overall screen-viewing we need to understand and then develop approaches to reduce multi-screen viewing among children.

## Background

Screen-viewing time (TV viewing, computer game time and internet use) has been associated with higher levels of adult obesity, type 2 diabetes and all cause mortality [1,2]. Screen-viewing has also been associated with increased body mass, increased risk of metabolic syndrome and lower psychological well-being among children and adolescents [3-6]. Youth screen-viewing is a relatively stable behaviour which tracks moderately from youth to adulthood [7], and high levels of youth TV viewing have been associated with increased risk of adult obesity [8]. Among adults participating in the US National Health and Nutrition Examination Survey, long periods of sitting time have been associated with adverse health outcomes while breaks in sitting time have been associated with lower waist circumference and C-reactive protein levels [9]. While there is currently a shortage of comparable data for youth it seems plausible that as well as screen-viewing overall sitting time may also be associated with adverse health outcomes among youth. Many national organisations have recognised the potential problems associated with high levels of youth screen-viewing and have implemented guidelines and health promotion campaigns that include a focus on strategies to reduce youth screen-viewing [10,11].

In order to develop effective interventions to change youth screen-viewing it is necessary to better understand the behaviour and the factors that influence whether a child is a high or low screen-viewer [12,13]. Traditionally, TV viewing has been the dominant form of youth screen-viewing and there is an extensive body of research that has reported on patterns of youth TV viewing [14,15], compliance with TV viewing recommendations [16,17] and associations between TV viewing and obesity [3]. In recent years the field has evolved with additional assessments of computer and console/video game time and other sedentary behaviours being included in childhood studies [18,19]. There is, however, a shortage of information about the nature of contemporary screen-viewing amongst children especially given the rapid advances in screen-viewing equipment technology and their widespread availability. For example, TV programmes are watched on computers, games consoles can be used to surf the internet, Smartphones, tablet computers and hand-held games play music, video games and provide internet access, and laptop computers can do all of the above. Anecdotal evidence suggests that large numbers of children embrace the multi-functionality of current devices to engage in multiple forms of screen-viewing at the same time but we are not aware of any study that has examined this issue. As such, we do not know whether multi-screen viewing takes place, if multi-screen viewing is associated with adverse health outcomes or if interventions that target multi-screen viewing should be developed. In addition, children's media multi-tasking may have implications for the measurement of screen-viewing and sedentary time. For example, if a child is asked to self-report the time they spend using different screen-viewing devices as individual questionnaire items this may lead to an over estimation of screen-viewing time if they are using multiple screens concurrently.

The aim of the current paper was to address current research limitations by conducting an exploratory, qualitative study in which we examined whether UK children report engaging in multiple forms of screen-viewing. We also specifically sought to examine where multiple forms of screen viewing occur, the nature of the viewing and the reasons for engaging in the behaviour.

## Methods

### Recruitment and Participants

Focus groups were conducted with 10-11 year old children who were recruited from five primary schools in Bristol, UK. To represent the spectrum of local economic diversity we recruited a primary school from each quintile of the Government's school deprivation indicator for Bristol schools [20]. All Year 6 (N = 261) students attended a brief presentation in which they were invited to take part in a research project. At the end of the presentation a member of the study team answered questions and provided information packs. The study was approved by a University of Bristol Ethics Committee. Written parental consent was obtained for 63 children (24% response rate). Ten focus group participant lists were generated by randomly selecting 3 boys and 3 girls as members of each group. Following participant drop out, 55 children (30 girls, 25 boys) participated in the focus groups, ranging in size from 4 to 6 pupils.

### Focus Groups

The focus groups were conducted in school classrooms and were recorded using a digital recorder (Olympus DS-3400). One team member facilitated the focus groups (SJS) while another took notes during the group (IHC). The focus group was based on a semi-structured topic guide. Topics included the types of screen-viewing in which the participants engaged; whether the participants ever engaged in more than one form of screen-viewing at any time and if so the nature of this multiple viewing; reasons for engaging in multi-screen-viewing; the room within the house where multi-screen-viewing took place and the reasons for selecting that room. The semi-structured format allowed for the discussion of new topics raised by participants and for nuances to be pursued. Focus groups were on average 44 minutes in duration (range = 25 to 60 minutes).

### Data analysis

All focus groups were transcribed verbatim and anonymised. As this project represented exploratory research and we had no pre-conceived ideas about the likely responses, we adopted a thematic analytical approach [21]. Firstly, the transcripts were read line by line by two team members (SJS and RJ) who marked the text with initial codes that described the content of the response [22]. Coding and theme generation was iterative and refined throughout analysis. Codes were entered as tree nodes in NVivo (Version 8, QSR, Southport, UK). Hierarchies of codes were created and summarised and themes within each group were developed and reviewed by all of the authors and interpretations were discussed and amended as necessary.

## Results

The analysis yielded two overall themes. The first theme was *Screen-viewing types and access to equipment *and explores children's current screen-viewing behaviours. The second theme was *Multi-screen-viewing*; and consisted of 3 sub-themes exploring the type and nature of multi-screen viewing, reasons for multi screen viewing and the locations and times of day that these behaviours take place. Themes and sub-themes are presented in detail below.

### 1) Screen-viewing types and access to equipment

In order to initiate a discussion about screen-viewing the focus group started by asking participants to identify the types of screen-viewing equipment that they had access to in their homes. Almost all of the children (96%) reported having access to a TV, 76% had access to a handheld console such as a Nintendo DS, DSi or a Sony PSP, 75% had access to a games console, 71% a laptop, 62% a PC and 51% an interactive mobile phone. The participants reported that on average they had five different screen-viewing devices within their home (range 3-8). The responses to this question therefore indicated that the participants had a number of opportunities to engage in multi-screen viewing.

In terms of activities, it was clear that TV viewing was very common with viewing including both the watching of TV programmes at their scheduled time and watching TV "on-demand" via the internet or recorded favourite programmes. For example:

*"Because you can get like iPlayer and just watch it live" *(Female)

*"I went camping with my friends on the day when it was the [reality show] final so I just went on 'TV on Demand' and I went on 'catch up' because they don't have the break. It's the same but they don't have the break, the adverts*" (Male) "

*If I watch [TV programme] and there's something brand new on like that's apparently really good and I want to watch that but they're on at the same time. Sometimes I record like the one that's new so I can watch [TV programme] so I can catch up with what's happening*" (Female)

The participants also reported extensive use of laptop computers and generic computers. It appeared that both devices were mainly used for social networking, online gaming, chat and watching videos on YouTube.

*"I'm on my DSi and my laptop. On my DSi I'm on MSN and on my laptop I'm on Facebook and then the TV is on" *(Female)

*"I chat to my friends on the laptop quite a lot" *(Male)

Participants also reported extensive used of handheld games consoles such as Nintendo DS, PSP and Smartphones which were mainly used as a medium to play games.

*"I play on the DS for hours on end" *(Female)

*"I play on my mum's iPhone all the time....just play on the games that are on there" *(Male)

*"I use my mobile phone to like text people and then I watch TV like before I go to bed" *(Female)

### 2) Multi-screen-viewing

The participants reported that they engaged in multi-screen viewing and further analysis indicated that this overall theme could be divided into: a) the forms of multi-screen viewing and the reasons for engaging in the behaviour; b) where multi-screen viewing took place, and the reasons for choosing that location; and c) when multi-screen viewing took place. Each sub-theme is presented below.

#### a) Multi screen-viewing forms and reasons for engaging in the behaviour

A number of children reported watching TV and using a laptop at the same time. For some children these dual behaviours were engaged in because the child was bored during TV commercials.

"*I watch TV and then I get bored with all the adverts so I get my laptop and start going on it and then when the show comes back on it I watch it, and then when it's the adverts I play on my laptop*" (Female).

"*So it's nice just when you're tired just to lounge back and watch the TV and then when it's adverts you just watch something else like YouTube*" (Female)

For many children the TV was just used as a method of filling time while they waited for a game to load on the laptop:

"*I was going to say if I am on the laptop, if like something is loading on the laptop I would watch TV while it is loading*" (Male)

"*When the computer is loading and a games takes ... and it's like 1%, 2% and it needs to get to 100% and it's loading for a long time then I just turn the TV on because I'm quite impatient*" (Female)

Children reported occasions when the TV was on but the programme was predominantly being watched by another family member. In these instances, TV viewing was a background behaviour with the majority of the attention focussed towards the laptop:

"*So he [father] was watching that and I just didn't want to watch it so I picked up my laptop and started going on my laptop, because I don't like [motoring programme] so you do something else*" (Female)

Finally, the children also reported using a laptop while watching TV as it enabled the children to do two things at once.

"*I think it's because I'm really eager to do both things, like when you're watching TV, I like to go and watch TV but I also really want to do something else on the computer at the same time*" (Male)

"*You might be watching like your favourite TV programme and if you were playing on something at the same time you might like ... say you were on a game and it seems to you that it's really important, like you've got to complete the levels so you've got to watch it at the same time*" (Female)

It was also clear that multi-screen-viewing occurred using a TV and a handheld console because either the child was not particularly interested in the TV programme that someone else was watching or the handheld was used to fill time during commercial breaks.

"*Sometimes I DS while my parents are watching the news or while my brother is watching something like [TV programme] or something like that*" (Female)

"*Yes on TV while I am waiting, while the adverts are going I just sometimes get a DS out or something*" (Male)

"I can't be bothered to change the channel so I just play on my DS" (Male)

Participants reported using mobile phones while either watching TV or using a computer. In these instances the phone use was either a function of responding to incoming calls and texts while engaged in TV viewing or a desire to engage in two behaviours at once.

"*Sometimes I do two things because like when somebody texts me and I text them back. It's like when the computer is loading again I just text somebody*" (Male)

"*I sometimes like watch the TV and go on my laptop, or like go on my phone and watch the TV*." (Female)

Similarly, handheld devices were often used as methods to fill time and address boredom when using a laptop.

"*Sometimes I go on my DS or something when a game is loading or something on my laptop... because I don't like waiting, I'm very impatient*." (Male)

Although the majority of the participants reported that they engaged in multi-screen viewing, and overall there were positive attitudes towards multi-screen viewing, there were a few female participants who did not enjoy or engage in multi-screen viewing.

*"It is easier to concentrate on one thing rather than, like, two things. Because if I try and do two things at once, I can't concentrate on what I'm doing" (*Female)

*"Well, I don't really do two screen things. I wouldn't use a DS or anything, I'd read" *(Female).

#### b) Multi screen-viewing location and reasons for location selection

The participants reported that multi-screen viewing occurred either in their bedroom or in the main living area of the home. The bedroom was the most frequently reported location for multi-screen viewing and this location was often selected because it was perceived as a quiet space that afforded privacy from siblings and adults.

*"In my bedroom I do the TV, the laptop and the music because that's my own place and I just relax in there so it's usually there" *(Male)

*"Mostly I do it in my bedroom because I like, having conversations with my friends and watching TV in private" *(Female)

*"I normally go upstairs, it's more like there's loads of people downstairs so it's somewhere quieter in my bedroom*" (Male)

The bedroom was also often used for multi-screen viewing because it was the room where the screen viewing equipment was stored.

"*I have a laptop in my bedroom and I have a TV in my bedroom and a DS and iPod and everything*" (Male)

"*In my room, because I've got a telly, a laptop and all the games consoles in my room apart from the Wii, which is downstairs*" (Female)

The main family area was the other frequently reported location for multi-screen viewing with this location selected because of the location of the equipment, the comfortable nature of the surroundings and the opportunity to spend time with other family members.

*"Well at my mum's I'd mostly be downstairs on the Xbox, but at my dad's I'm normally downstairs because he's got a 32" flat screen telly on the wall so I just like chill out and just watch TV" *(Male)

*"I go in the living room because it is like really relaxing...you have got like the windows open, the sun is coming in and it is just nice and pleasant" *(Male)

"*I would normally do it downstairs because it is with family*" (Female)

"*And like in the living room it is nice to have a bit of company sometimes*" (Female)

#### c) Timing of multi-screen viewing

The participants reported that multi-screen viewing occurred across the day but the main periods were after-school and at the weekend. It was, however, noticeable that there was considerable variability in the level and timing of viewing and this appeared to be a function of whether the participants attended after-school clubs with club or activity participation resulting in lower levels of screen-viewing after school.

### After school and evenings

Many children explained how their multi-screen viewing would start once they arrived home from school.

*"When I come home from school, your friends want to tell you something but they don't want to tell you in school so you go on Facebook and you talk to them" *(Female)

*"Normally about four o'clock after school because, that's when all the repeats are on after school as well so I normally go on the laptop or on the phone or something like that" *(Female)

*"When I go home from school I sit on the sofa and watch TV for like an hour and a half or something, then I go up to play on my PlayStation 2. Then my brother comes in and annoys me and says, 'I want to play with you,' and then I have to, go and get my DS so then we link up or play with the DS. Then, like after half an hour of that, um, I go back on my PlayStation and then, um, send a few text messages on my phone to my friends" *(Male)

A number of participants indicated that their screen-viewing would persist through the evening until they went to bed.

*"Sometimes I do watch it all the way to bedtime" *(Male)

*"I have tea and then me and my family just sit down and watch TV...I take my PSP up to bed and just play on that until like twelve o'clock" *(Male)

Some children identified that going to after school activities or clubs prevented them from screen-viewing in the home.

*"I don't do it on the weekends or Thursday because on Thursday I've got my piano lessons and on the weekend I just like to get out at weekends because it's free days, you don't have to go to school" *(Female)

"On school days I usually have a club so when I get home I'm usually just ... I don't get much time to watch TV. So on weekends I get more time" (Male)

"I only do it on weekends usually after school, I do quite a lot of stuff like I do football and then I do ... like I go swimming and stuff like that and go around my friend's house"

### Weekends

Some participants reported greater screen-viewing at weekends and this was perceived to be a function of the increased time available.

*"At the weekends I watch more telly because there's more time to fill. When you're at school you're only home before it's another four hours before you ... another six hours before you go to bed... at weekends usually people are busy so there's more time to watch telly because you don't have other things to do" *(Male)

*"On the weekends when I first get up I play on my DS and then erm after breakfast I go on the computer till lunch. Then I go back on, then when tea comes I go eat it and then I play on my DS till I go to sleep*" (Female)

In contrast, other participants' weekends were full of activities outside of the home which they associated with having less time for screen-viewing:

"So at weekends I've got loads of things on so I don't watch TV, like almost not at all". (Male)

"Well on Saturday, I do a club so in the morning me and my sister wouldn't go on anything until about three o'clock because the clubs are so long, so at three o'clock I'd probably then go on the laptop or maybe go out with my friends" (Female)

### Mornings

Some participants also reported engaging in multi-screen viewing before school but this appeared to be a less prevalent behaviour.

*"In the morning I play on my DS till my mum gives me breakfast and after I get dressed if I have got time" *(Female)

*"Like, when I'm, like, in bed, just wake up, turn on the TV, and go on the laptop" *(Female)

## Discussion

Consistent with previous studies the data presented here suggest that contemporary UK children are engaged in high amounts of screen-viewing [6,23]. Although there was considerable variability in viewing patterns, screen-viewing appears to be an embedded behaviour with many children regularly engaging in two or more forms of screen viewing at the same time. It is important to highlight that although the majority of the multi-screen viewing involved TV viewing, this rarely appeared to be the dominant activity. This finding might be a function of the passive nature of TV as it is possible to do another activity while watching TV. It is, for example, more difficult to do something else whilst playing a high action, fast response video game. The data reported here, therefore, suggest that new strategies that focus on reducing multi-screen viewing are required.

Participants reported that there were three main reasons for engaging in multi-screen viewing. Firstly, it tempered impatience that was associated with a programme loading or waiting for a response to a text message or instant message. For these children the second or third screen filled the time and prevented boredom. Secondly, multi-screen viewing was a reactive response that enabled the child to use their time more efficiently as they could filter out unwanted content such as advertisements and focus their attention on just the content that interested them. Thirdly, multi-screen viewing was a proactive decision with the children opting to do two or more things at once as it was perceived to be more interesting or more enjoyable. It is not clear the extent to which the proactive viewing was influenced by high levels of access to multiple pieces of screen-viewing equipment or a more general preference. Either way, as the children reported having access to an average of five pieces of screen-viewing equipment in their home it is relatively easy for the majority of children to engage in multi-screen viewing. Collectively, these findings suggest that in order to reduce screen-viewing we need to be able to improve our understanding of why children engage in multi-screen viewing. The increased understanding could then be used to inform the development of intervention approaches that are targeted towards the underlying factors that influence an individual child's viewing behaviours.

### Implications for strategies to reduce youth screen-viewing

Screen-viewing, and multi-screen viewing in particular, occurred either within the main family room or within the child's bedroom. Moreover, a considerable proportion of the screen-viewing occurs on portable devices that can easily be moved from one room to another within the house. Previous studies have indicated that the presence of a TV in a child's bedroom has been associated with higher screen-viewing [24]. Consequently a number of intervention approaches have focussed on removing TV's and media equipment from children's bedrooms as part of broader screen-viewing reduction strategies [25]. The portable nature of current devices suggests that such approaches may not be appropriate for contemporary viewing patterns. Alternative approaches that focus on limiting screen-viewing time, regardless of location, may be more relevant for contemporary viewing patterns. These new approaches could also be mapped onto goal setting and goal monitoring interventions in which the children take ownership of their own viewing behaviours.

Participants reported extensive use of on-demand TV-viewing facilities. It is not clear if the use of on-demand services resulted in a higher volume of viewing as the children could always find something of interest to watch, or if the on-demand services enabled the participant to only watch programmes of particular interest without an increase in volume. A number of interventions that have focussed on reducing screen-viewing have included goal setting components in which the participants have been encouraged to identify the programmes that they want to watch and then limit their viewing to those programmes [25,26]. On-demand services could therefore be a useful tool that facilitates TV viewing goal setting and monitoring. As such, further research that specifically focuses on: a) whether on-demand viewing results in higher overall viewing levels; and b) whether use of on-demand services can be used as a goal-setting tool to reduce overall screen-viewing time, is warranted.

The after-school period appears to be a critical period for screen-viewing. After-school screen-viewing appears to be influenced by whether the child engaged in organized after-school activities with club participation associated with lower screen-viewing. Although the data presented here are not objective, the patterns reported are consistent with after-school physical activity literature. A number of studies have suggested that the period between the end of the school day and the evening meal is when discretionary physical activity occurs [27,28]. The after-school period is also when the highest proportion of moderate-to-vigorous physical activity is obtained [29]. As such the after-school period has been identified as a critical window for the promotion of physical activity and a number of interventions have focussed on increasing physical activity at this time [28,30]. The data presented here suggest that the after-school period may also be a key time period in which to limit screen-viewing and strategies that focus on reducing screen-viewing during this window are needed.

### Measurement

Measuring sedentary behaviours for purposes of both research and population surveillance is challenging. Our findings raise some important issues for assessment. Given the clear indication that children both switch between screen tasks on a frequent basis, and that multi-tasking on screens also occurs, the way we assess such behaviours needs a closer look. There are two main outputs from assessing sedentary behaviours. These are total sedentary time, best assessed using objective methods such as accelerometers or posture monitors, and time spent in key sedentary behaviours, such as screen time. Self-report measures will be used for this. Assessing individual sedentary behaviours is necessary if we are to better understand the behavioural patterning of certain sedentary tasks and for the development of behaviour change interventions.

Using objective methods for assessing total sedentary time will be unaffected by the discovery that many young people are likely to be multi-tasking on screens. However, these behavioural patterns do have important implications for assessing individual behaviours by self-report. For example, it is possible that children switching between TV viewing and laptop use, or operating them more or less simultaneously, may over-report time in both when responding to certain questions. Self-report questions may need to address this by allowing the reporting of multi-tasking or for stating the main task being undertaken. Assessment of sedentary behaviour will also be affected by the development of new technologies that allows for 'convergence' of platforms. For example, children already watch TV programmes or films on their laptops, and listen to music played from Smartphones. The implications of such changes for measurement are yet to be explored but it is an issue for consideration.

### Strengths and limitations

This is the first study to assess the extent to which children engage in multi-screen viewing and as such the paper advances our understanding of youth screen-viewing behaviours. We recognise that quantitative data from a larger number of participants would be needed to ascertain the extent of youth engagement in multi-screen viewing. However, the information included in this paper is an essential first phase of research and could be used to inform the development of more extensive assessment. Although we recruited schools to provide participants from a range of socio-economic backgrounds the sample is not sufficiently large to allow for a formal analysis of differences by socio-economic group. The major limitation of the work is the relatively small sample of participants that were recruited from five schools in a single city which limits our ability to generalise findings to other cities and countries.

However, it was clear that saturation, where no new information was obtained from subsequent student focus groups, was achieved. We are therefore confident that the data presented here are an accurate representation of the views of children in the local area.

## Conclusions

The data from this qualitative study indicate that contemporary UK children regularly engage in two or more forms of screen-viewing at the same time. As such there is a need to assess multi-screen viewing and associations between multi-screen viewing and health outcomes. However, there are currently no means of assessing multi-screen viewing and therefore techniques that assess multi-screen viewing are urgently needed. Equally, there are currently no interventions that specifically focus on reducing multi-screen viewing. A greater understanding of why multi-screen viewing occurs, how on-demand services are used, and how multi-screen viewing could be reduced in the after-school period is needed to design new, more effective interventions. Multi-screen viewing is a new form of screen-viewing and in order to reduce children's overall screen-viewing we need to understand and then develop approaches to reduce multi-screen viewing.

## Competing interests

The authors declare that they have no competing interests.

## Authors' contributions

The project was conceived by RJ, SJS, TG and SJHB. All data were collected by SJS and IH. Analysis was performed by SJS, RJ and IH. RJ led the drafting of the manuscript with all authors adding sections for the paper. All authors made critical contributions to the manuscript and approved the final version.
